# Training in Diagnostic Hysteroscopy: The “Arbor Vitae” Method

**DOI:** 10.3390/medicina59061019

**Published:** 2023-05-24

**Authors:** Ivan Mazzon, Andrea Etrusco, Antonio Simone Laganà, Vito Chiantera, Silvia Di Angelo Antonio, Valentina Tosto, Sandro Gerli, Alessandro Favilli

**Affiliations:** 1 Arbor Vitae Endoscopic Centre, 00191 Rome, Italy; i.mazzon@arborvitae.it (I.M.); silvia.diangeloantonio@gmail.com (S.D.A.A.); 2 Unit of Gynecologic Oncology, ARNAS “Civico—Di Cristina—Benfratelli”, Department of Health Promotion, Mother and Child Care, Internal Medicine and Medical Specialties (PROMISE), University of Palermo, 90127 Palermo, Italy; etruscoandrea@gmail.com (A.E.); antoniosimone.lagana@unipa.it (A.S.L.); vito.chiantera@unipa.it (V.C.); 3 Centre for Research in Perinatal and Reproductive Medicine, University of Perugia, 06123 Perugia, Italy; tosto.valentina@libero.it; 4 Section of Obstetrics and Gynecology, Department of Medicine and Surgery, S. Maria della Misericordia Hospital, Perugia University, 06123 Perugia, Italy; sandro.gerli@unipg.it

**Keywords:** hysteroscopy, training model, diagnostic hysteroscopy, questionary, simulator

## Abstract

*Background and Objectives*: Diagnostic hysteroscopy is the gold standard in the diagnosis of intrauterine pathology and is becoming an essential tool in the daily practice of gynecology. Training programs for physicians are necessary to ensure adequate preparation and learning curve before approaching patients. The aim of this study was to describe the “Arbor Vitae” method for training in diagnostic hysteroscopy and to test its impact on the knowledge and skills of trainees using a customized questionnaire. *Materials and Methods*: A three-day hysteroscopy workshop combining theory and practical “hands on “sessions with dry and wet labs has been described. The aim of the course is to teach indications, instruments, the basic principles of the technique by which the procedure should be performed, and how to recognize and manage the pathologies that can be identified by diagnostic hysteroscopy. To test this training method and its impact on the knowledge and skills of the trainees, a customized 10-question questionnaire was administered before and after the course. *Results*: The questionnaire was administered to 34 participants. All trainees completed the questionnaire, and no missing responses were recorded. Regarding the characteristics of the participants, 76.5% had less than 1 year of experience in performing diagnostic hysteroscopy and 55.9% reported performing fewer than 15 procedures in their career. For 9 of the 10 questions embedded in the questionnaire, there was a significant improvement in the scores between pre- and post-course, demonstrating a perceived significant improvement in theoretical/practical skills by the trainees. *Conclusions*: The Arbor Vitae training model is a realistic and effective way to improve the theoretical and practical skills required to perform correct diagnostic hysteroscopy. This training model has great potential for novice practitioners to achieve an adequate level of proficiency before performing diagnostic hysteroscopy on live patients.

## 1. Introduction

Diagnostic hysteroscopy is the gold standard in the evaluation and diagnosis of uterine cavity morphology and intrauterine pathologies [1]. With high sensitivity and specificity, high feasibility, and low complication rates, diagnostic hysteroscopy is a minimally invasive procedure that can be performed in an outpatient setting without anesthesia [2]. Nowadays, diagnostic hysteroscopy should completely replace blind endometrial biopsies such as dilation and curettage [3,4,5].

When performed with the correct technique, hysteroscopy is well tolerated by patients and the success rate reported in the literature ranges from around 90 to 95% [6]. Conversely, the incorrect application of this procedure, due to suboptimal technical skill, can cause discomfort/pain [2,7,8] and even serious complications [9].

Considering the wide range of indications, hysteroscopy has become a fundamental tool in the daily practice of gynecology. Therefore, the adequate performance of diagnostic hysteroscopy should be considered a basic skill for all gynecologists [10]. Training programs with the objective of giving theoretical notions and technique principles are necessary in order to adequately prepare physicians and, above all, to provide an effective learning curve before approaching patients [11,12]. 

In the last decade, several models have been proposed for training in hysteroscopy. In order to reproduce the anatomy of the uterus and the characteristics of its tissues as accurately as possible, simulators based on vegetables and animal models, as well as synthetic and virtual models, have been designed and described [12,13]. 

Since 1995, the Arbor Vitae group has been organizing a three-day hysteroscopy workshop combining theoretical lessons and hands-on practice sessions, applying preparatory exercises with dry and wet labs. The course objective is to teach indications, instruments, the basic principles of the technique by which the procedure should be performed, and how to recognize and manage the pathologies that can be identified by diagnostic hysteroscopy.

The aim of this study was to describe the Arbor Vitae method for training in diagnostic hysteroscopy, articulated in theoretical, video, and “hands on” practical sessions, and to test its impact on the knowledge and abilities of trainees using a customized questionnaire.

## 2. Materials and Methods

### 2.1. Training Course Structure

#### 2.1.1. Theoretical and Video Session

In the theoretical and video session, basic principles, instruments, and techniques to correctly perform diagnostic hysteroscopy are explained. 

The understanding of diagnostic hysteroscopy cannot be separated from a good knowledge of the instruments and their correct usage to perform the procedure with the correct technique. The first approach to the procedure takes place during the theoretical sessions. The applicability of each instrument component is illustrated with pictures and videos, with special attention and focus on the vision provided by the 30° optics. In addition, the anatomy of the cervix and the uterine cavity, the pathologies that can be diagnosed, and the possible complications that can occur are also shown and described. A short constructive debate between students and trainers is held at the end of each lecture. 

During the video session, videos of diagnostic hysteroscopies are presented and discussed, showing the endoscopic view of the physiological condition of the cervix and uterine cavity, associated pathologies, and correct and incorrect procedures.

##### Uterine Cervix: How to Overcome It

The cervix is the “door” to the uterine cavity and, in a sense, the hysteroscopist’s “tomb” at the same time, so knowing how to overcome it is essential to a successful procedure. Therefore, the assessment of the cervical canal, its description, and correct navigation are of a particular importance. Pictures and videos are used to teach the trainee how to assess the basic characteristics of the cervical canal such as caliber, direction, morphology, cervical mucosa, and vascularization. The correct technique for navigating the cervical canal is also shown, with particular emphasis on how to take advantage of the 30° fore-oblique optical system. The trainee should learn that, as far as the execution technique is concerned, an incorrect angle of the instrument could cause pain, lesions of the cervical mucosa, perforation, bleeding, or a wrong path. Failure to navigate properly within the cervix may result in a failed examination. 

The video sessions show the trainee how to proceed along the cervical canal, keeping the image of the lumen itself at 6 o’clock, following the course of the folds of the arbor vitae, the course of the cervical vessels, and the flow of the distending medium. Respecting the structures of the endocervix is essential for the success of the procedure. In case of doubt or difficulty in advancing the instrument, the trainee is shown how to stop the hysteroscope and move it backwards. The distending medium will then show the correct way to continue the procedure.

##### Uterine Cavity: How to Explore It

Once the cervix has been overcome, the 30° fore-oblique view continues to be the basis for the study of the uterine cavity, thus avoiding tilting movements which, as in the case of the cervix, could cause avoidable pain. The rotation movements around the longitudinal axis of the optical system allow the examination of the tubal ostia (90° rotation) and the anterior and posterior walls of the uterus (180° rotation), providing a complete evaluation of the uterine cavity, an effective identification of any pathology, and a fast procedure with minimal movement and high patient comfort.

#### 2.1.2. Hands-on Session

The aim of the practical “hands on” session is to make the trainee confident with the instruments and how to assemble them, how to navigate the cervix correctly and how to examine the uterine cavity using the 30° fore-oblique view. The trainee must learn to use the three basic movements of hysteroscopy: translation, rotation, and swing, overcoming the “fulcrum effect”, which is a typical mistake made by beginners when approaching the endoscopic view. Trainees are divided into groups of three at each station, and each group has a tutor to guide them through the exercises. Each station is equipped with a table with a fluid collection sheath, an “all-in-one” Tele-Pack+ Storz system (Karl Storz, Tuttlingen, Germany), which includes a monitor, light source, and full HD camera control unit with integrated network function in a single compact mobile unit, an ENDOMAT distension liquid pump (Karl Storz, Tuttlingen, Germany), and a 5 mm hysteroscope (Bettocchi hysteroscope, Karl Storz, Tuttlingen, Germany).

The first step of the hands-on session is to become familiar with the instruments. The hysteroscope provided to the trainees is composed of a Hopkins optical system of 2.9 mm with a 30° fore-oblique view, an internal sheath with a diameter of 4.3 mm equipped with an operative channel for semi-rigid surgical instruments, and an external sheath with a diameter of 5 mm for the outflow of the liquid distension medium (Bettocchi hysteroscope, Karl Storz, Tuttlingen, Germany). The tutor demonstrates how to assemble and disassemble the hysteroscope and how to make the connections to the monitor, the light source, and the distension liquid pump. Then, in order to develop a tactile sensitivity to the instrument, the trainee is invited to assemble and disassemble the hysteroscope, first under direct vision and then blindly. 

**Figure 1:**
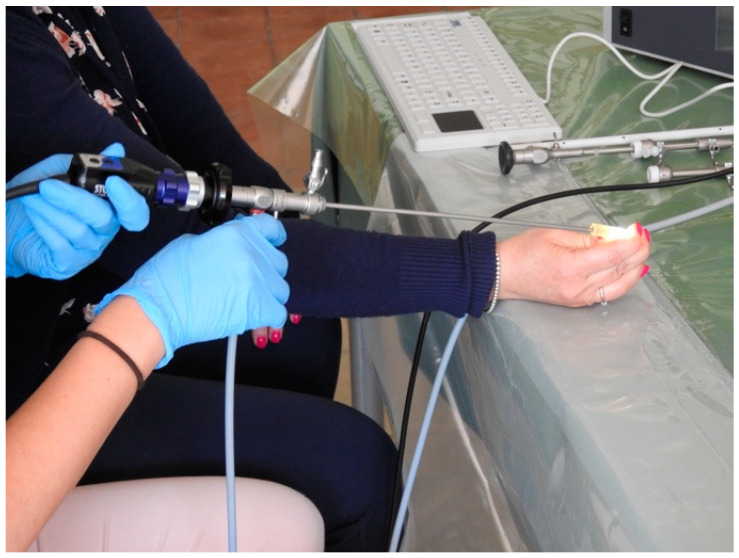
Trainee navigates the rubber tube by putting into practice the 6 o’clock vision.

Once the learners have become familiar with the fundamentals of the instruments, they begin exercises to develop or improve the 30° fore-oblique view. This step begins with basic exercises: the students are provided with longitudinal rubber tubes of standard caliber and are asked to navigate them by putting into practice the 6 o’clock view illustrated during the theoretical and video sessions. Although much simpler than crossing the cervix, the rubber tube immediately provides a very clear and realistic way to learn the correct positioning of the instrument within the canal and provides adequate tactile perception when the hysteroscope is correctly navigated (Figure 1).

**Figure 2:**
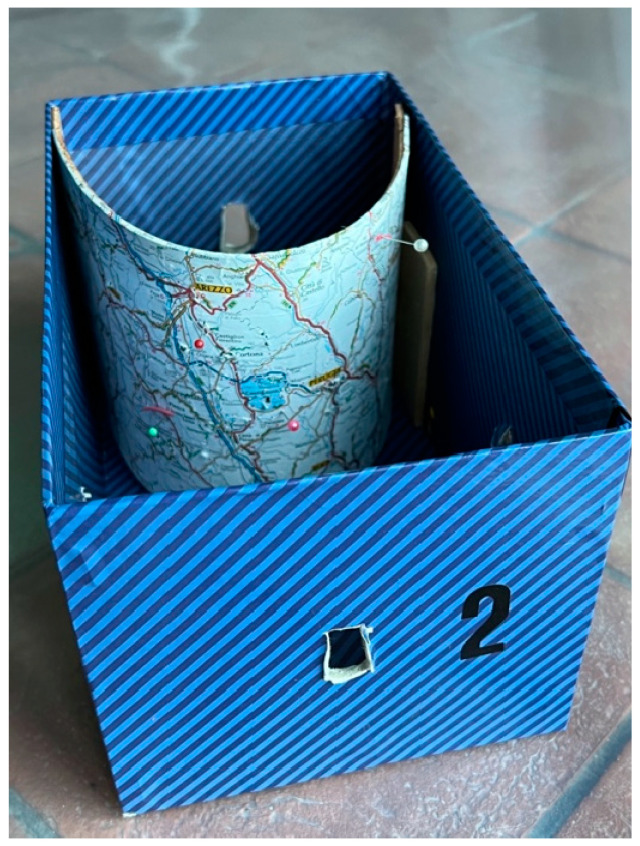
Cardboard box and curved map with pins.

**Figure 3:**
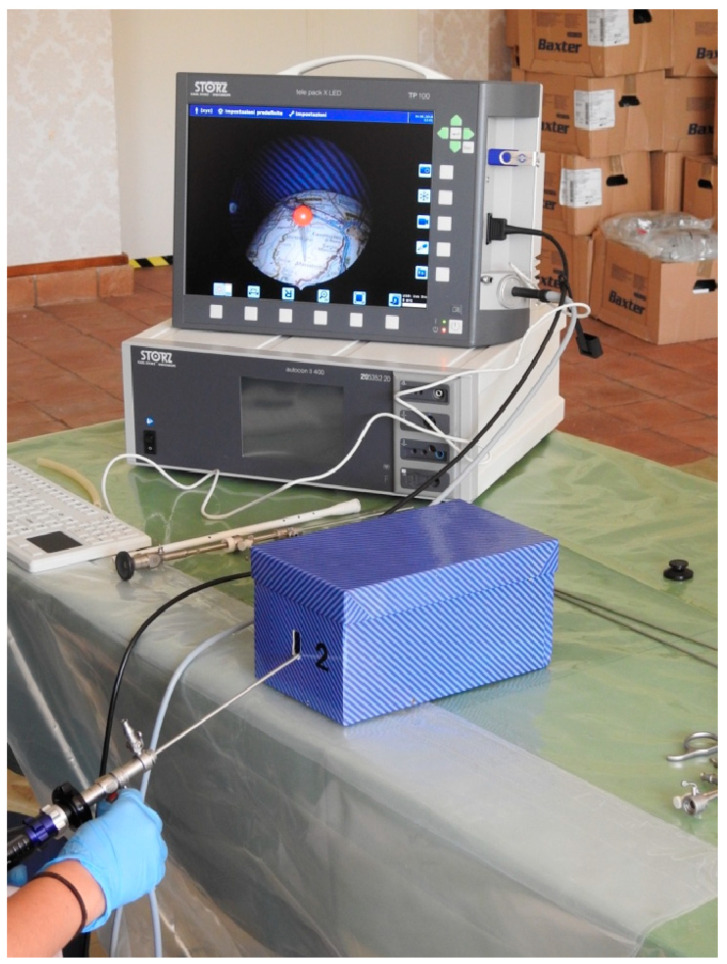
Trainee practices on the cardboard box.

The second step is to use a cardboard box with a road map in a convex structure in the fundus and a hole through which the instrument can be inserted. This exercise aims to help the trainee to cope with the 30° fore-oblique view along to the basic movements of hysteroscopy (translation, rotation, and swing), as in the uterine cavity. The map presents pins at various locations (Figure 2). The tutor proposes different itineraries to be followed, starting from two nearby destinations connected by a linear path and ending in more complex paths. The student’s task is to follow the road that connects two different points on the map, with the aim of placing the road image in the centre of the monitor, taking advantage of the perspective offered by the 30° view and, thus, trying to reduce the movements of the hysteroscope (Figure 3).

**Figure 4:**
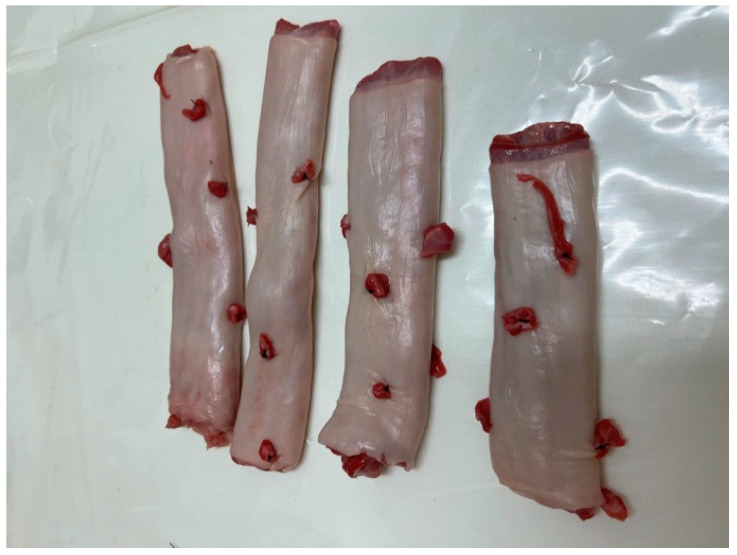
Rumen before packaging with the piece of flesh sutured on it.

**Figure 5:**
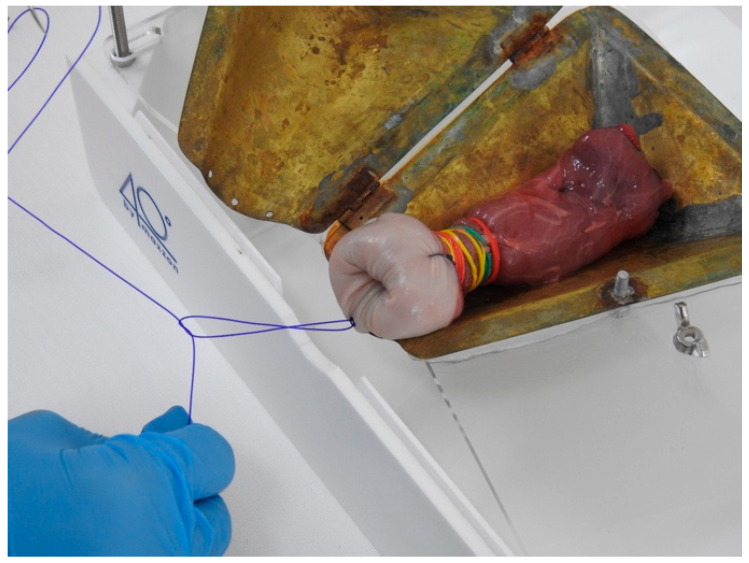
Animal model of our course and the rigid plastic box.

The third step is based on exercises using a biological model. The womb model used for this course is an animal model: a cow’s rumen. To simulate endometrial polyps or leiomyomas of the uterus, the rumen is arranged with pieces of animal flesh of different consistencies sewn into it and then closed at one end with a suture (Figure 4). The open end is then turned over and tightened with numerous rubber bands to simulate the cervical canal and the isthmus, like the human one. The packed rumen is placed on a closed metal support, which allows it to expand; the whole item is then fixed to a rigid plastic box with an entrance hole, which is supplied to each station (Figure 5).

The trainees are then invited to perform a real diagnostic hysteroscopy, putting into practice all of the skills acquired during the theoretical and video sessions and the previous practical exercises. After assembling the hysteroscope and making all connections, the trainee inserts the hysteroscope into the external orifice of the rumen under direct vision, realistically simulating the passage into the cervical canal. The trainee must keep the image of the cervical lumen fixed at 6 o’clock and try to move the instrument backwards if the path to follow is not clear. Once inside the cavity, the trainee should proceed with a systematic evaluation as follows: first, the two tubal ostia should be investigated by rotating the hysteroscope through 90° on its axis; then, the anterior and posterior walls should be evaluated by rotating it through 180° on its axis (see Video S1). Finally, the trainee should try to become familiar with the tactile sensation of touching the anatomical structure with the tip of the hysteroscope.

### 2.2. Trainee Improvement Testing by Questionnaire

In order to evaluate the effectiveness of our diagnostic hysteroscopy training method, the trainees were asked to complete the same questionnaire at the start and at the end of the course in order to assess their feelings of improvement. The questionnaire consisted of ten questions, each scoring from 0 (minimum) to 10 (maximum), designed to assess the theoretical knowledge and technical–procedural level of the trainees in diagnostic hysteroscopy. The outcome assessors were blind to the identity of each respondent.

The design, analysis, interpretation of data, drafting, and revisions conform to the Helsinki Declaration, the Committee on Publication Ethics guidelines and the Strengthening the Reporting of Observational Studies in Epidemiology Statement [14], available through the Enhancing the Quality and Transparency of Health Research Network. The data collected were anonymized, taking into account the observational nature of the study, without personal data that could lead to the formal identification of the participants. Each participant enrolled in this study signed consent to allow data collection and analysis for research purposes. The study was not advertised. No remuneration was offered to the participants to give consent. As this research involved normal educational practices in the context of a training course, this study was exempted from Institutional Review Board approval.

Statistical analysis was performed with InStat 3.10, GraphPad Software, San Diego, CA. Continuous variables were expressed as mean and standard deviation (SD), or median and interquartile range (IQR), as appropriate. Categorical variables were expressed as frequency and percentage. The independent t-test and Wilcoxon rank-sum test were used to compare continuous variables as appropriate. The χ2 test and Fisher’s exact test were used to compare categorical data. A *p*-value < 0.05 was considered statistically significant.

Since the questionnaire was customized and used for the first time in this study, we had no previous data to use as a base for the sample size calculation. Nevertheless, in hypothesizing a mean score of 5 ± 2 before the start of the course (average for a medium knowledge of the topic), and an expected 20% increase in the overall score after the course, the enrolment of 31 participants would achieve a power of 80% with an alpha error of 0.05.

## 3. Results

The questionnaire was administered to 34 participants (10 residents and 24 specialists in gynecology and obstetrics). All trainees completed the questionnaire, and no missing responses were recorded. 

The complete characteristics of the participants are described in Table 1. Twenty-six course participants (76.5%) had less than one year of experience in performing diagnostic hysteroscopy, and six participants (17.6%) had already participated in at least one previous diagnostic hysteroscopy course. Moreover, 19 participants (55.9%) reported performing fewer than 15 procedures in their career. 

For 28 participants (82.4%), the diagnostic hysteroscopies were carried out in an outpatient setting, while 6 participants (17.6%) performed the procedures in the operating room with sedation.

The 10 questions given before and after the course all use a scale from 0 to 10 and all of the results are shown in Table 2. For 9 out of the 10 questions, there was a significant improvement (*p* < 0.0001 for questions 1, 2, 3, 4, 6, 9, and 10; *p* = 0.0025 for question 5; *p* = 0.0002 for question 7) of the scores between the pre- and post-course. The only question for which there was not a significant improvement was n° 8: “From 0 to 10, how do you judge the accuracy of diagnostic hysteroscopy with endometrial biopsy as compared to dilation and curettage?”, where the mean pre-course score was 9.1 and the post one was 9.44 (*p* = 0.3628). 

## 4. Discussion

In comparison to other endoscopic procedures, diagnostic hysteroscopy, when performed correctly, is a well-tolerated procedure that allows the evaluation of the uterine cavity without anesthesia on an outpatient basis [8,10,15]. However, these characteristics of high safety and efficacy may lead some gynecologists to mistakenly believe that it is a procedure that can be performed without adequate training. Such a misguided attitude could be responsible for many hysteroscopies being performed with the wrong technique, causing discomfort and avoidable pain to the patient, complications, and inconclusive procedures [2,7,15,16]. One of the main causes of failure in diagnostic hysteroscopy is probably the innate reflex of the novice endoscopist to aim at the centre of the screen when navigating the cervical canal [17,18]. In fact, the failure to master the 30° fore-oblique view determines the patient’s pain and thus the failure of the procedure.

The percentage of gynecologists who still perform diagnostic hysteroscopy in a questionable way, who are not familiar with the endoscopic view, and who use incorrect methods to gain access to the uterine cavity may still be high. Therefore, there is a need for training programs that aim to provide theoretical concepts and practical principles to perform the correct technique and to provide adequate training to gynecologists [12,13].

The Arbor Vitae method, through the intensive combination of theoretical, video, and hands-on practical sessions, aims to improve the knowledge of diagnostic hysteroscopy and practical skills to correctly perform the procedure. These principles are fundamental to developing a good technique and performing the diagnostic hysteroscopy correctly. 

Several training programs using different simulators have been described and reported: plant simulators have been proposed using butternut pumpkin [19], animal organs such as bovine uterus [20] or pig bladder [21], or even virtual models [18,22,23]. All of these have different characteristics, but the aim is to provide the trainee with an easy way to simulate the uterus in order to gain confidence in the use of the hysteroscope before approaching a live patient. The Arbor Vitae method is characterized by the variety of models used in the practical sessions, starting with preparatory exercises on simple plastic tubes to familiarize the student with the instruments and their correct use in the cervix; then, using a card box, the trainee consolidates the movements to be used when examining the uterine cavity. Finally, all that has been learned is tested on an animal model. This step-by-step progressive pathway allows the trainee to gradually and effectively develop the 30° fore-oblique view, while becoming familiar with the correct movements and how to minimize them and developing tactile perception by using the tip of the hysteroscope.

Another important feature of our training method is the realism and reproducibility of our simulator. The rumen, as packaged in these courses, allows trainees to practice repeatedly and develop the right feel and perception with the instruments during the procedure in a highly realistic way. 

The low cost of the proposed models is another aspect to consider. In fact, the cost of rubber tubing, cardboard boxes, and rumen is negligible. The only additional cost is the cost of keeping the rumen at a low temperature in the fridge, a cost that can be accepted by almost all centers. The most important expense of our course concerns the instruments. Providing workstations with modern, complete, and fully functional instruments could be expensive. Training programs were also proposed with rudimentary or self-assembled equipment—for example, a smartphone instead of the camera we used—supporting the advantage of lower cost [24]. 

The use of the questionnaire allowed us to test the impact of the knowledge and skills of the trainees. The significant improvement in theoretical and practical knowledge of diagnostic hysteroscopy shown by the mean scores in 9 out of 10 questions, even for the most experienced participants, suggests the effectiveness of the method. Nevertheless, several limitations should be taken into account for proper data interpretation: first of all, the number of enrolled participants was low, although it fits the sample size analysis; secondly, we used a customized questionnaire, without any previous validation; thirdly, the participants were not blinded to the aim of the questionnaire administration, so they may have given higher scores on purpose after the course to manifest their satisfaction. Considering these elements, we solicit further studies in order to confirm our preliminary findings in a large cohort analysis.

## 5. Conclusions

Diagnostic hysteroscopy has become a basic skill that every gynecologist should have in order to meet the demands of daily clinical practice. In the era of precision medicine, the need for training courses in diagnostic hysteroscopy, well-structured and effective in transmitting theoretical and practical notions is increasingly, especially for those who make minimally invasive their working philosophy.

The Arbor Vitae method aims to provide a solid foundation for gynecologists wishing to perform diagnostic hysteroscopy and to critically evaluate each procedure. Thanks to the realism and reproducibility of the models used, the trainee learns the basics of navigation and assessment of the cervical canal and uterine cavity, while becoming familiar with the diagnostic procedure and the instruments required.

The data from the questionnaire are very encouraging and demonstrate the effectiveness of the Arbor Vitae training method. The participants reported a significant improvement in both theoretical and practical skills when using this training approach for diagnostic hysteroscopy. This training model has great potential for novice practitioners to achieve an adequate level of proficiency prior to performing diagnostic hysteroscopy on live patients. However, further research designed on the perception of live patients undergoing diagnostic hysteroscopy is needed to validate the effectiveness of the Arbor Vitae training method in improving the performance of trainees.

## Figures and Tables

**Table 1:** Characteristics of the included participants.

	Participants (*n* = 34)
Age, years (mean ± SD)	41.6 ± 9.9
Females *n* (%)	24 (70.6)
Males *n* (%)	10 (29.4)
Residents *n* (%)	10 (29.4)
Specialists *n* (%)	24 (70.6)
Years of profession (mean ± SD)	10.4 ± 9.1
**Years of diagnostic HSC experience:**	*n* (%)
<1	26 (76.5)
1–5	7 (20.6)
>10	1 (2.9)
Previous HSC courses (%)	6 (17.6)
**N° of diagnostic HSC**	*n* (%)
<15	19 (55.9)
15–30	4 (11.8)
30–50	2 (5.9)
>50	9 (26.5)
**N° of diagnostic HSC per session**	*n* (%)
<3	19 (55.9)
From 3 to 6	9 (26.5)
From 6 to 10	6 (17.6)
>10	0
**N° diagnostic HSC per week**	*n* (%)
<5	23 (67.6)
From 5 to 10	8 (23.5)
>10	3 (8.8)
**Usual setting**	*n* (%)
Outpatient	28 (82.4%)
Outpatient + anesthesia	0
Operating room	6 (17.6%)
Technical difficulty encountered (0 = never; 10 = always) (mean ± SD)	4.94 ± 2.1
Failure of the procedure (0 = never; 10 = always) (mean ± SD)	3.74 ± 2.6
Pain caused to the patient (0 = no pain; 10 = maximum conceivable pain) (mean ± SD)	4.75 ± 3

Data are expressed as mean ± standard deviations for continuous variables, or as percentages for dichotomous variables. HSC: hysteroscopy.

**Table 2:** Results of the customized questionnaires administered before and after the course.

	Pre-Course	Post-Course	*p*
Question 1: How do you consider your knowledge of the indications for diagnostic hysteroscopy? (0 = no knowledge; 10 = perfect knowledge)	7.15 ± 2.1	8.82 ± 1	<0.0001
Question 2: How do you judge your knowledge of the instrumentation used in diagnostic hysteroscopy? (0 = no knowledge; 10 = very good knowledge)	4.6 ± 2.4	8.44 ± 1.1	<0.0001
Question 3: How do you consider your skills in recognizing hysteroscopic pictures? (0 = not at all confident; 10 = very confident)	5.2 ± 2.3	7.15 ± 1.3	<0.0001
Question 4: How do you judge your confidence level in the use of the 30° fore-oblique view? (0 = not at all confident; 10 = very confident)	4.6 ± 2.3	7.82 ± 1.1	<0.0001
Question 5: How do you consider your ability in navigation and assessment of the cervical canal, according to a scale from 0 to 10? (0 = not at all confident; 10 = very confident)	5 ± 4	7.27 ± 1.3	0.0025
Question 6: Express your level of confidence in the management of complications in diagnostic hysteroscopy, using a scale from 0 to 10 (0 = not at all confident; 10 = very confident):	4 ± 2.6	7.18 ± 1.5	<0.0001
Question 7: From 0 to 10, how much do you consider diagnostic hysteroscopy useful before performing an operative hysteroscopy?	8.5 ± 1.7	9.74 ± 0.6	0.0002
Question 8: From 0 to 10, how do you judge the accuracy of diagnostic hysteroscopy with endometrial biopsy as compared to dilation and curettage (0 = no utility; 10 = maximum utility)	9.1 ± 1.2	9.44 ± 1.8	0.3628
Question 9: From 0 To 10, give a general judgment on what you consider is your current level of theoretical knowledge of diagnostic hysteroscopy	5.7 ± 1.9	8.32 ± 1.3	<0.0001
Question 10: From 0 To 10, express a general judgment on what you consider is your current level of technical-procedural skills in diagnostic hysteroscopy	4.3 ± 2.3	6.76 ± 1.3	<0.0001

Data are expressed as mean ± standard deviations.

## Data Availability

Not applicable.

## Supplementary Materials

The following supporting information can be downloaded at: https://www.mdpi.com/article/10.3390/medicina59061019/s1, Video S1: Endoscopic view of the animal model.
